# Indoor Positioning Systems: A Blessing for Seamless Object Identification, Monitoring, and Tracking

**DOI:** 10.3389/fpubh.2022.804552

**Published:** 2022-02-23

**Authors:** Shilpa Shyam, Sujitha Juliet, Kirubakaran Ezra

**Affiliations:** Karunya Institute of Technology and Sciences, Department of Computer Science and Engineering, Coimbatore, India

**Keywords:** indoor positioning systems (IPS), technique and technology, challenges, features, industry 4.0

## Introduction

Technology is the greatest result of supreme human imagination. The continual proliferation of indoor positioning technology is laid out in this paper along with its advantages and challenges. The authors point out that there are more to be grasped and utilized in this powerful and growing domain.

## The Prominence of Indoor Positioning Systems in the Past, Present, and Years to Come

The global navigation satellite system (GNSS) performs exceedingly well in finding accurate location data anywhere on the planet. It is most sought after for its high accuracy and global coverage. The efficiency of GNSS is only dominant outdoors due to heavy signal multipath and signal attenuation. However, it fails to meet expectations for indoor environments, which is why several indoor localization technologies have popped up. Indoor navigation systems can be wearables, wall mounted devices, or an intelligent model able to calculate the precise location of objects or humans in any sort of sophisticated indoor environment backed up with several obstacles. An indoor navigation system consists of three vital modules: 1. Indoor positioning system module to estimate the object position, 2. Navigation module which helps in routing the object from the current destination, and 3. Object interaction module which helps in providing instructions to the model or system (1). The three module system results in better localization and navigation (modeling, surveying, and mapping of infrastructures) of location-based assets or object tracking, especially in emergency services for disaster management. With the daily invention of new applications, this industry is expected to have a market value of about 24 billion dollars by the year 2023. The aviation industry makes use of this system in helping passengers navigate to lounges, track passenger baggage, and perform other airport related security services. The advertising industry utilizes location-based promotions for the E-commerce sector. The healthcare sector implements location-based services for tracking patient records and whereabouts within the hospital arena. Asset or object-based tracking using the three module system is an inevitable part of the logistics industry. Through this positioning technology, customers can be traced and helped in navigating toward various services available in a railway station, bus stands, etc., benefiting the transportation industry. Indoor positioning technology has also seen a surge in the tourism and automotive industries as easy navigation of tourists and their assets can be monitored along with vehicle identification. The next time one visits the Sydney airport, one could witness the use of apple maps in which navigating through each terminal is made easy using this technology. Indoor positioning technologies not only comply with commercial sector standards but are also made available for day-to-day home services and applications. A tango augmented reality-based indoor location technology has been developed by the technology giant, Google. It would provide detailed and precise location data of the user using their mobile device. Apple has reached far ahead with indoor positioning technologies. They have employed inbuilt ultrawide band (UWB) chips in premium IPhones to calculate the location of a user in real time.

## Salient Techniques and Technologies in Indoor positioning System

The basic principle behind the indoor navigation and positioning system is to accurately measure the range and distance between two devices. This can be done in two basic methods. The first one is the measurement of the distance using received signal strength (RSS), in which the strength of the signal between the transmitter and receiver determines the location. Though the accuracy is found to be considerable, it is highly influenced and affected by multipath propagation. Conventional but superior technologies, including WiFi, Bluetooth, RFID, Dead Reckoning, Ultrasonic, and ZigBee, fall under this category.

Radio Frequency Identification (RFID) avails the use of radio waves for object detection. The RFID readers and tags undergo interchanging of frequencies during this process. An RFID-based tracking system was implemented for dynamic targets with <1 m localization accuracy which proved it to be a propitious feature for applications where tracking is needed (2). Peer-to-peer communication over shorter distances can be easily established using the most common Bluetooth technology. ZigBee is a sought-after technology when a low cost and low power system has to be implemented. This makes it suitable to be implemented it in smart homes where energy conversation is taken care of Tumlin (3). Dead Reckoning, unlike other technologies, contemplates velocity for measuring position. It determines the present location based on velocity and past position data. A smartphone-based pedestrian dead reckoning system evinced the need for further implementation in this arena by providing exceptional results in indoor positioning systems (4). In ultrasonic systems, the distance is computed using the time of arrival between the emitter and receiver. The coordinates of the emitter are assessed using multilateration to the fixed anchors. The second measurement involves the estimation of the time of flight from several devices. This method comparatively imparts centimeter accuracy and is used by the UWB technology. UWB utilizes both the time difference of arrival (TDOA) and time of arrival (TOA) for measurement purposes. It is also seen to play a significant role in the industrial revolution 4.0.

Several smart factories have emerged by inculcating UWB (5). Khan et al. (6) defend various wireless technologies, including Wi-Fi and LoRa, to be the most worthy of implementing indoor localization applications because of it being vigorous, affordable, and able to utilize a minimum amount of power.

Technologies and techniques in indoor positioning systems go hand in hand. Combinations of technologies or combinations of techniques are perceived to be infused for better accuracy in recent times. Techniques in indoor positioning can be separated into triangulation, proximity, fingerprinting, and vision analysis. The computation of asset location using geometrical features of triangles is known as triangulation. It is further used for computation in two ways: lateration and angulation. Lateration measures distance alone for positioning, unlike angulation which uses both angles and distances. Fingerprinting is conducted in two stages, the online stage (also known as the serving stage) and the offline stage (also known as the training stage) for precise object calculation. Vision analysis is carried out from images received from several points. When an object is detected with respect to a known position, it is known as proximity analysis and requires several fixed detectors for this purpose.

## Selection of Relevant Techniques and Technology According to the Environment and Need of the Hour

There is a huge disparity found in the requirements of indoor positioning when compared to outdoor systems. The dissimilarity in requirements is due to the diverse layout in indoor environments as they have complicated and sophisticated pathways. Hence, the accuracy and coverage demand would vary accordingly. Indoor positioning systems built specifically for assisted living, monitoring patients at home, etc., have a requisite for accuracy within 1 m, whereas systems operated for urban and rural applications demand accuracy of about a few meters. Thus, keeping in mind the application type and its place of execution, a suitable technology has to be chosen. As there is a need for technology in all sectors, no indoor position system can be claimed as the ideal solution (7). Along with accuracy and coverage, maintenance and implementation cost, system size, and power consumption are essential metrics.

The design of an indoor positioning system commences in two stages. First, by determining the principle indoor positioning technology on which it would be based upon, and second, by determining the technique that would be infused along with it. Systems that are supposed to create smart homes, find objects that are misplaced, and track and monitor daily activities are usually implemented using ZigBee, WiFi, and fingerprinting (8). Bluetooth has been adapted for low cost and low power applications (9). Similarly, ZigBee devours minimal power and is inexpensive in most cases and is, thus, used for home applications. Applications that require huge coverage area and centimeter accuracy within larger areas, including industries and manufacturing sites, preferably implement UWB (10). They are capable of imparting huge amounts of data using minimal energy. Tracking the motion of a visually impaired person or the movement of humans within a small area can easily be implemented by pedestrian dead reckoning, which is an example of dead reckoning technology (11). Newborn systems based on indoor positioning have been seen to work when using aerial robots, mobile robots (12), and humanoid robots. In such cases, criteria such as battery efficiency and power consumption are vital. Based upon the technology that was chosen to be executed, the suitable technique would be integrated according to the environment and the need of the application. Table 1 puts forth a comparison of the various technologies used at present in terms of accuracy, range, power consumption, and noise tolerance.

**Table 1:** Comparison of existing indoor localization technologies.

**Technology**	**Accuracy**	**Range (m)**	**Power consumption**	**Noise tolerance**	**Disadvantages**
UWB(Ultra wide band	Very high	1–50	Low	Very high	UWB signal are prone to get obstructed by huge objects.
technology)					
WiFi	Low	1–50	High	Medium	Utilizes ISM band interferences.
Bluetooth	Medium	1–20	Low	Medium	Suffers from low range
RFID	High	1–50	Low	Medium	Communication security is a question. Suffers from low coverage
ZigBee	Very high	1–50	Low	Medium	Lowe rate of transmission
Dead Reckoning	Medium	1–100	High	Medium	Require high quality sensors

## Prevailing Challenges in the Implementation of Indoor Localization Systems

Every technology under the indoor positioning system is considered supreme, but it brings inexorable challenges along with it. Once the basic technology and technique are made obvious, the challenges that come with it must be tackled without compromising the requirements of the system. The impediments in an indoor environment should be considered and precision in location data should be accurate (13). Contemporary research proves that UWB technology is largely used for industries and manufacturing sites where it could track and trace both static and dynamic objects at ease. Though its utility is large, the signals of UWB are easily hindered by the indoor obstacles, thereby making error mitigation a necessity (14). Conventional methods such as WiFi and Bluetooth are often considered less often as it offers low range. Ultrasound is often neglected when used for wide ranging locations and has frequency restrictions. Privacy and security are one category that is often abandoned while considering metrics in indoor positioning systems (15). These systems are customized to provide accurate locations of data to the user and its organization alone. The involvement of a third party in such systems is a threat to the user or the organization responsible. Hence, future research is expected to pay more attention to the privacy and the security content of the indoor positioning systems along with the security of the data of users.

## Discussion

The needs of humans and technology are swiftly changing. Such needs should acknowledged to bring about changes and revolutions over the course of time. Indoor positioning technology is one such domain that attends to the needs of humans in several ways. Hence, researchers are always on the lookout for new formulations in this arena. Every technology under this system is beneficial and addresses particular complications. It is up to the researcher or the industrialist to select the appropriate technology and technique according to the application needs. From healthcare to travel, indoor positioning technologies are universal and omnipresent. With the internet of things (IoT), intelligent systems and mobile computing are growing at a fast pace as the market of indoor positioning technology has been dramatically increasing. Despite its several advantages, it also comes with several challenges for researchers to improve on. Particularly, the metrics of indoor location systems are its premier challenges. In addition, accuracy, maintenance cost, coverage, scalability, and privacy are major challenges that need to be subdued by implementing efficient measures. Finally, special heed should to be given to the privacy and security of the indoor positioning systems for the personal privacy and security of users.

## Author Contributions

SS conceived the concept and drafted the manuscript. SJ supervised the study and verified the manuscript. KE performed the review and editing. All authors contributed to the article and approved the submitted version.

## Funding

This study was supported by the Department of Science and Technology-Natural Resource Database Management System (DST-NRDMS) [Grant No: NRDMS/UG/NetworkProject/e-13/2019 (C) P-4].

## Conflict of Interest

The authors declare that the research was conducted in the absence of any commercial or financial relationships that could be construed as a potential conflict of interest.

## Publisher's Note

All claims expressed in this article are solely those of the authors and do not necessarily represent those of their affiliated organizations, or those of the publisher, the editors and the reviewers. Any product that may be evaluated in this article, or claim that may be made by its manufacturer, is not guaranteed or endorsed by the publisher.
